# Peroxisome proliferator-activated receptor alpha is involved in the temperature-induced sex differentiation of a vertebrate

**DOI:** 10.1038/s41598-020-68594-y

**Published:** 2020-07-15

**Authors:** Seiji Hara, Fumiya Furukawa, Koki Mukai, Takashi Yazawa, Takeshi Kitano

**Affiliations:** 1 Department of Biological Sciences, Graduate School of Science and Technology, Kumamoto University, Kumamoto, 860-8555 Japan; 2 Department of Biochemistry, Asahikawa Medical University, Asahikawa, Hokkaido 078-8510 Japan

**Keywords:** Differentiation, Reproductive biology

## Abstract

Medaka (*Oryzias latipes*) is a teleost fish with an XX/XY sex determination system, similar to that of mammals. However, under high temperature conditions, XX medaka is masculinised by elevation of cortisol, the major teleost glucocorticoid. In this study, to identify novel factors in the gonads acting downstream from cortisol during sexual differentiation, we performed RNA sequencing (RNA-seq) analysis using the gonadal regions of larvae reared at normal temperature with and without cortisol, and at high temperature. The RNA-seq and real-time PCR analyses showed that expression of some peroxisome proliferator-activated receptor α (PPARα) signalling-targeted genes was increased by cortisol. PPARα agonist treatment induced masculinisation of XX medaka in some cases, and co-treatment of the agonist with cortisol further induced masculinisation, whereas treatment of *pparaa* knockout medaka with cortisol or the agonist did not induce masculinisation. This study provides the first evidence that PPARα is involved in environmental sex determination in vertebrates.

## Introduction

Many vertebrates have males and females that reproduce sexually. Sex is genotypically determined in many species, but sex determination is greatly affected by ambient temperature in poikilothermic vertebrates, including reptiles, amphibians, and fish^1^. Furthermore, environmental factors, such as pH^2^, density^3^, and social factors^4^, are suggested to override genetic sex determination in some teleost fish. However, the molecular mechanisms of environmental sex determination in these species remain poorly understood.

Medaka (*Oryzias latipes*) is a small teleost fish that offers many advantages, including the availability of many useful strains^5^. Transgenic (Tg) techniques and the gene knockout systems using clustered regularly interspaced short palindromic repeats (CRISPR)/CRISPR-associated protein 9 (Cas9), are established^6–8^. Moreover, the sex-determining gene *dmy* (also known as *dmrt1bY*) on the Y chromosome has been identified in this fish^9^. Therefore, medaka is a useful animal model for vertebrate genetic analysis of the sex determination and differentiation. The first appearance of morphological sex differentiation in medaka is the difference in the number of germ cells before hatching, as germ cells in genetic females (XX) undergo a rapid proliferation and subsequently initiate oogenesis while they remain quiescent in genetic males (XY)^10,11^. High temperature (HT) treatment during the sex differentiation inhibits germ cell proliferation and oocyte development in XX medaka, and causes masculinisation^12–14^. HT also causes masculinisation through elevating the levels of cortisol, the major teleost glucocorticoid^15^. Furthermore, exposure of XX medaka to cortisol or HT increases expression of *gonadal soma-derived growth factor* (*gsdf*) and decreases expression of *cytochrome P450 family 19 subfamily A member 1a* (*cyp19a1a*) by 5 days post-hatching (dph)^16^. However, it remains unclear how HT-elevated cortisol induces the masculinisation of XX medaka.

Cortisol is one of the major glucocorticoids secreted by the interrenal tissues, and its production is induced by various stressors, including the acidic water and rapid temperature changes^17^. Glucocorticoids act on target tissues by binding to the glucocorticoid receptors that operate as transcription factors and regulate a variety of systems, including neuronal function, ion regulation, growth and reproduction^18,19^. Recently, it has been reported that glucocorticoids appear to induce masculinisation not only in many fish species, such as pejerrey (*Odontesthes bonariensis*), Japanese flounder (*Paralichthys olivaceus*), and three-spot wrasse (*Halichoeres trimaculatus*)^20–22^, but also in the lizard species, which have a temperature-dependent sex determination system^23^. Therefore, glucocorticoids may have the potential to cause masculinisation in poikilothermic vertebrates.

Here, to identify novel factors in the gonads acting downstream from cortisol during sexual differentiation, we first performed RNA sequencing (RNA-seq) analysis using the gonadal regions of larvae reared at normal temperature with and without cortisol, and at HT. Next, we analysed the localisation of peroxisome proliferator-activated receptor α (PPARα) because we confirmed that cortisol increased the expression of some PPARα signalling-targeted genes using RNA-seq and real-time PCR analyses. Moreover, we examined effects of PPARα agonists and then generated *pparaa* knockout medaka and investigated its phenotypes.

## Results

### Transcriptome analysis in the gonadal region at hatching

**Figure 1:**
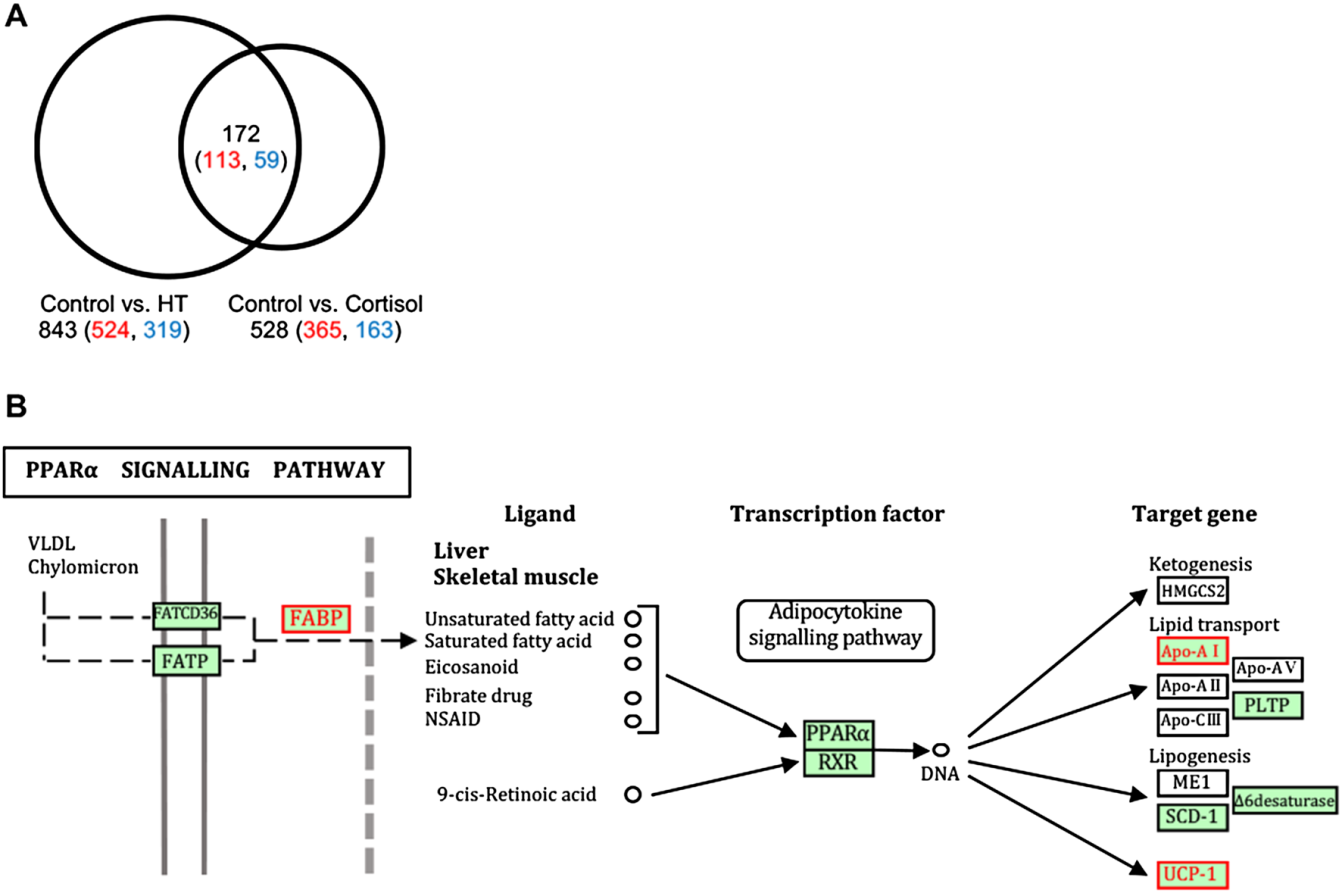
High temperature- and cortisol-induced changes in gene expression. (**A**) Venn diagram showing the number of differentially expressed genes (DEGs). Each value represents the number of DEGs, with the red numbers representing the number of up-regulated DEGs and the blue numbers representing the number of down-regulated DEGs. The numbers inside overlapping circles represent the number of common DEGs. (**B**) PPARα signalling pathway. Genes indicated by red boxes and text represent genes that match DEGs. FABP: Fatty Acid Binding Protein, Apo-AI: Apolipoprotein A1, UCP-1: Uncoupling Protein 1. This pathway drawn is mainly referred to the KEGG database^53–55^.

To identify novel factors induced by HT and cortisol in larvae gonads during sexual differentiation, we performed a comprehensive expression analysis using RNA-seq. RNA-seq analysis was carried out using 0-dph larvae treated with HT or cortisol, and the fragments per kilobase of exon per million mapped fragments (FPKM) value was calculated. Differentially expressed genes (DEGs) were defined as genes whose FPKM values were significantly changed under different experimental conditions. HT produced 843 DEGs and cortisol treatment produced 528 DEGs compared with control conditions. Of these DEGs, the number of genes whose expression was increased by both HT and cortisol treatments was 113, and the number of genes whose expression was decreased by both treatments was 59 (Fig. 1A). Of the common DEGs, up-regulated 77 DEGs and down-regulated 47 DEGs were categorized based on gene ontology (GO) annotations using the Blast2GO software. The DEGs included many apolipoproteins, and GO terms related to lipid metabolism were detected (Supplementary information Table S1). Furthermore, we used the KEGG Mapper to represent the distribution of DEGs in each medaka signalling pathway. As a result, *fatty acid binding protein 7* (*fabp7*), *apolipoprotein A1* (*apoa1*) and *uncoupling protein 1* (*ucp1*), were assigned to the PPARα signalling pathway (Fig. 1B). In addition, other up-regulated DEGs included *hsp70* and immune-related genes (*complement C5*, *complement C9*, *complement factor I*), but not sex differentiation-related genes (*gsdf*, *cyp19a1a*, *cyp19a1b*, *foxl2*, *amh*, *amhr2* and *dmrt1*) and glucocorticoid receptors (*gr1* and *gr2*).

### Expression pattern and localisation of PPARα

**Figure 2:**
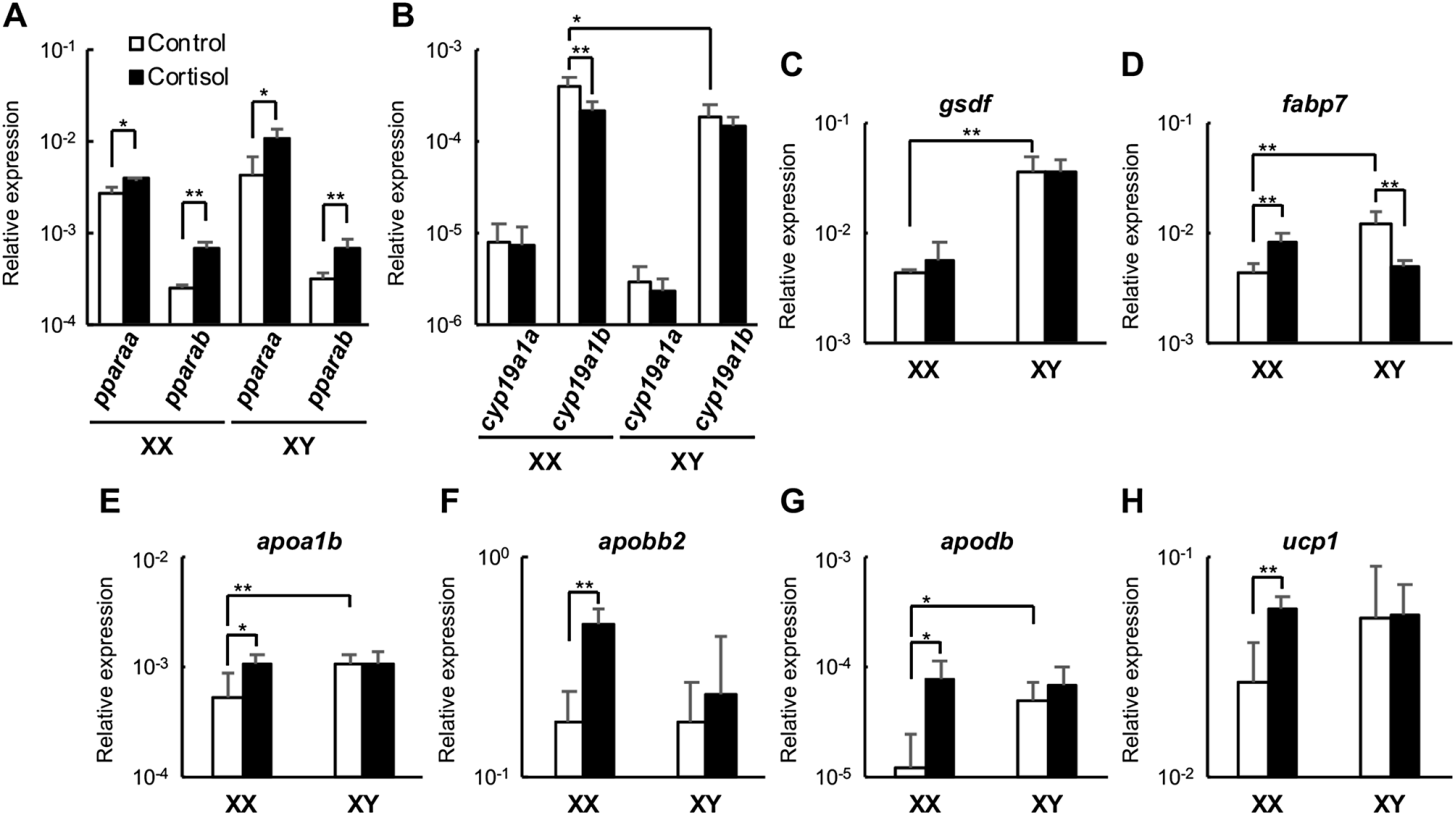
Cortisol increases the level of gene expression in the PPARα signalling pathway. Analysis of the expression of each gene in the gonadal region of 0-dph medaka by quantitative real-time PCR. Relative expression levels of the target genes were normalised to that of *ef1α*. (**A**) *pparaa* and *pparab*, (**B**) *cyp19a1a* and *cyp19a1b*, (**C**) *gsdf*, (**D**) *fabp7*, (**E**) *apoa1b*, (**F**) *apobb2*, (**G**) *apodb*, (**H**) *ucp1*. *p < 0.05, **p < 0.01, n = 5.

**Figure 3:**
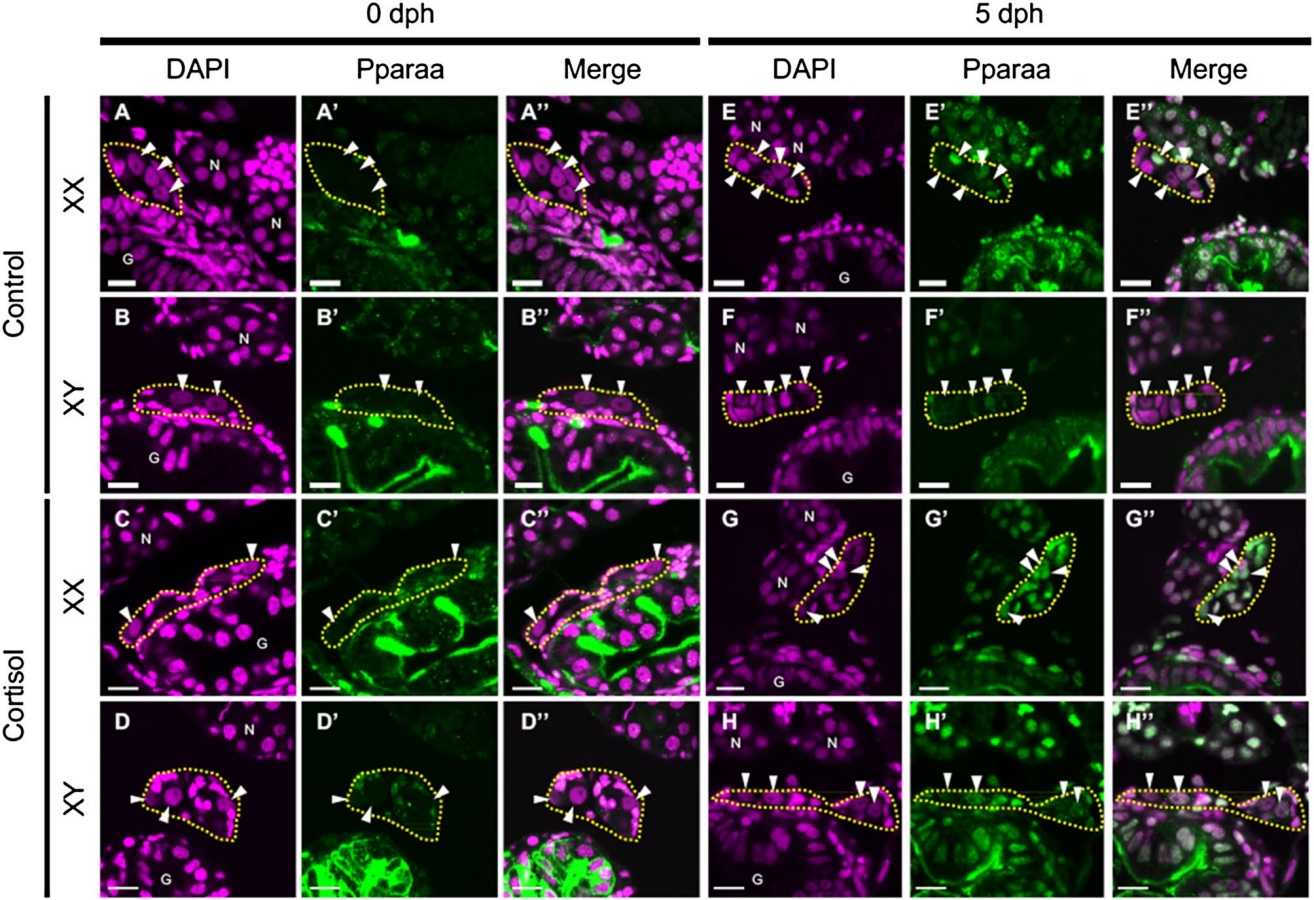
Cortisol induces the expression of Pparaa in gonadal somatic cells and germ cells. Immunofluorescence staining using an anti-Pparaa antibody. The gonadal regions of (**A**–**D**) 0-dph larvae and (**E**–**H**) 5-dph larvae. N: Nephridium, G: Gut, dotted circle outlines: gonads, arrowheads: germ cells. Scale bars: 10 μm.

Next, we confirmed the expression pattern of PPARα signalling-related and sex differentiation-related genes by quantitative real-time PCR. Cortisol significantly increased the expression levels of *pparaa* and *pparab*, genes encoding two PPARα subtypes, but the expression level of *pparab* was generally lower than that of *pparaa* (Fig. 2A). As for the genes encoding estrogen synthetase, *cyp19a1a* expression levels were not significantly changed by cortisol in either sex, whereas *cyp19a1b* had higher overall expression levels than *cyp19a1a*, and cortisol significantly decreased expression levels in XX individuals (Fig. 2B). The expression level of *gsdf* did not vary with cortisol in either sex (Fig. 2C). We then examined whether the PPARα signalling pathway is activated by cortisol. *fabp7*, apolipoprotein genes, *apoa1b*, *apobb2*, and *apodb*, and the mitochondrial uncoupling protein gene, *ucp1*, are all involved in this pathway and were significantly elevated by cortisol in XX individuals (Fig. 2D-H). Next, to examine the localisation of Pparaa, we carried out immunofluorescence staining of gonads using an anti-Pparaa antibody. Pparaa-positive signals were detected in the nuclei of many somatic cells, including gonadal somatic cells, in both XX and XY medaka at 0 dph, and the signals were stronger in cortisol-treated larvae than in control fish larvae (Fig. 3A–D). No signal was detected in germ cells at 0 dph. Similar experiments with medaka at 5 dph produced signals in the nuclei of gonadal somatic cells and germ cells in control larvae, which were stronger in cortisol-treated larvae (Fig. 3E–H). Pparaa positive signals in adult fish were detected in many follicles and somatic cells in the ovary, in germ cells such as spermatogonia and spermatocytes in the testis, and in nuclei of somatic cells, such as Leydig and Sertoli cells (Supplementary information Fig. S1). No signal in any sample was detected using rabbit normal serum (data not shown). Western blot analysis was used to further examine the proteins recognized by the anti-Pparaa antibody in medaka tissues. A single band displaying the Pparaa protein was detected in the testis and liver (Supplementary information Fig. S2).

### Effects of PPARα agonists on sexual differentiation

**Figure 4:**
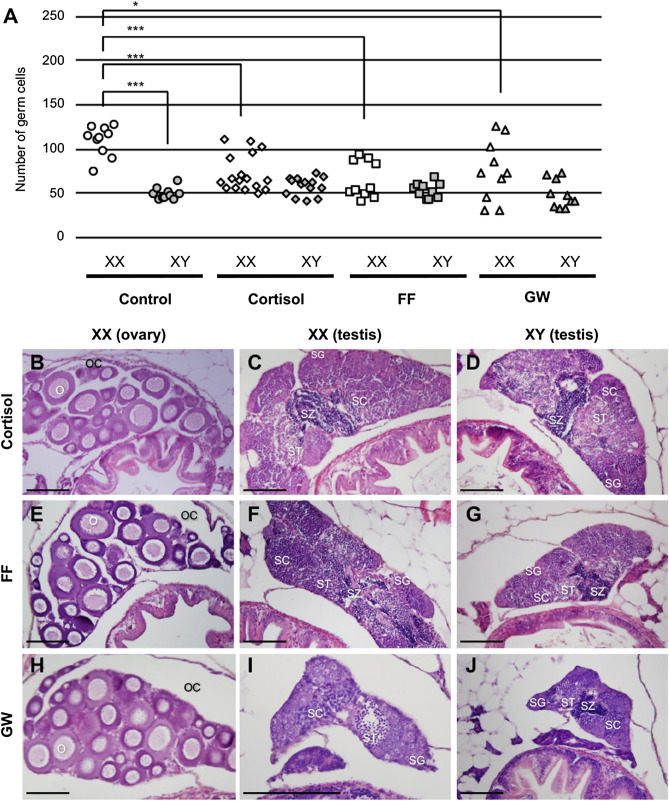
PPARα agonists induce masculinisation. (**A**) Number of medaka germ cells at 0 dph. *p < 0.05. ***p < 0.001. Gonads of adult medaka at 2 mph after (**B**–**D**) cortisol, (**E**–**G**) FF treatments and (**H**–**J**) GW treatment. O: oocytes, OC: ovarian cavities, SC: spermatocytes, SG: spermatogonia, ST: spermatids, SZ: spermatozoa. Scale bars: 100 μm.

To ascertain whether PPARα has a masculinising effect similar to that of cortisol treatment, we treated medaka with the PPARα agonists, fenofibrate (FF) and GW-7647 (GW), during the sexual differentiation period. These drugs have been used as PPARα activators not only in mammals but also in teleosts^24–26^. First, we confirmed activation of medaka Ppar-mediated transcription by FF and GW treatments using a luciferase assay. Both FF and GW significantly increased the activation when transfected with Pparaa, but not with Pparab, Pparb and Pparg (Supplementary information Fig. S3). Next, we counted the number of germ cells at the hatching stage in medaka treated with FF, GW and cortisol from 0 days post-fertilisation (dpf). Approximately half of XX individuals treated with FF or GW had approximately 50 germ cells, corresponding to the number in male types, as did those treated with cortisol (Fig. 4A). We then examined the sex ratio of adult fish by histological observation of the gonads at 2 months post-hatching (mph) after treatment from 0 to 5 dph. Low dose FF treatment did not induce masculinisation of XX medaka, whereas high dose FF and GW induced masculinisation in some cases (Fig. 4B–J, Table 1). Moreover, co-treatment of the agonists and cortisol further increased the rate of masculinisation (Table 1). Histological examination showed that all XY and the sex-reversed XX males had normal testes with productive spermatogenesis, but not intersexes (Fig. 4B–J).

**Table 1:** Sex ratios in adult medaka.

		No. of adult fish				% of
Genotype	Treatment	XY♂	XY♀	XX♂	XX♀	XX sex-reversal
wild-type	Control	13	0	0	9	0
	Cortisol	37	0	7	25	21.9
	1 μM FF	24	0	0	37	0
	5 μM FF	16	0	2	6	25.0
	5 μM FF + Cortisol	11	0	7	7	50.0
	1 μM GW	9	0	1	6	14.3
	1 μM GW + Cortisol	8	0	7	6	53.8
*pparaa*^*-/-*^	Control	11	0	0	12	0
	Cortisol	8	0	0	10	0
	5 μM FF	13	0	0	11	0

### Generation of *pparaa* knockout medaka and its phenotype

**Figure 5:**
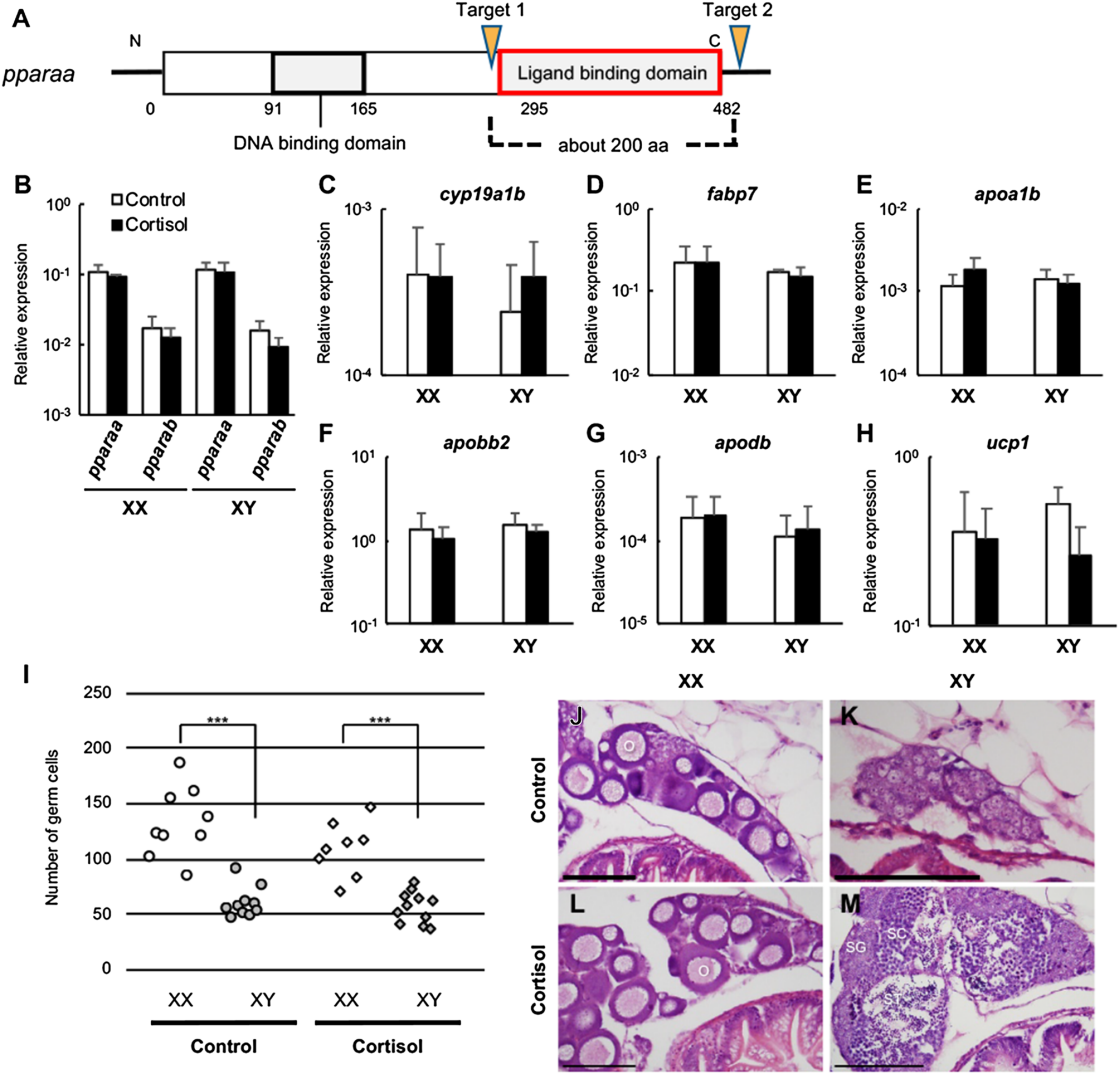
*pparaa*^-/-^ medaka are not masculinised by cortisol. (**A**) A schematic representation of the exon sequence of medaka *pparaa* and the target site of crRNA. (**B**–**H**) Quantitative real-time PCR analysis of the expression of each gene in the gonadal region of 0-dph *pparaa*^-/-^ medaka. Relative expression levels of the target genes were normalised to that of *ef1α*. (**B**) *pparaa* and *pparab*, (**C**) *cyp19a1b*, (**D**) *fabp7*, (**E**) *apoa1b*, (**F**) *apobb2*, (**G**) *apodb*, (**H**) *ucp1*. n = 5. (**I**) The number of germ cells in *pparaa*^-/-^ medaka at 0 dph. ***p < 0.001. (**J**–**M**) Gonads of *pparaa*^-/-^ adult medaka. O: oocytes, SC: spermatocytes, SG: spermatogonia, ST: spermatids, SZ: spermatozoa. Scale bars: 100 μm.

To determine whether *pparaa* is an essential gene for cortisol-induced masculinisation, we knocked out medaka *pparaa* using the CRISPR/Cas9 system^8^. As shown in Fig. 5A, the ligand-binding domain was eliminated by the simultaneous cleavage of the *pparaa* sequence by two CRISPR RNAs (crRNA). This was performed following the example of the PPARα null mouse^27^. Loss of the ligand-dependent function of Pparaa was achieved by simultaneously injecting two crRNAs, a trans-activating crRNA (tracrRNA) and Cas9 protein into medaka embryos at the 1-cell stage. The genotype of *pparaa* was determined by the length of the PCR product amplified from genomic DNA. *pparaa* knockout fish (*pparaa*^-/-^) were obtained by crossing heterozygous mutants. PPARα signals in *pparaa*^-/-^ medaka were examined by quantitative real-time PCR to determine whether cortisol activates PPARα signals and whether *cyp19a1b* expression is altered by cortisol. In *pparaa*^-/-^ medaka, neither PPARα signal-related genes nor *cyp19a1b* were significantly altered by cortisol at hatching (Fig. 5B–H). Next, the effects of cortisol and PPARα agonist on *pparaa*^-/-^ medaka was confirmed. When the number of germ cells at hatching was counted, there was no inhibitory effect by cortisol on germ cell proliferation in *pparaa*^-/-^ medaka (Fig. 5I). Examination of the sex of adult fish showed that *pparaa*^-/-^ medaka were not masculinised by cortisol and FF (Fig. 5J–M, Table 1).

## Discussion

In this study, to identify novel factors acting downstream from cortisol in the gonads during sexual differentiation, we performed RNA-seq analysis using the gonadal regions of larvae treated with HT or cortisol. The expression of genes related to fatty acid metabolism was changed significantly under both HT and cortisol conditions, and KEGG mapping analysis indicated activation of the PPARα signalling pathway. Moreover, real-time PCR analysis revealed that cortisol increased the expression levels of *fabp7*^28^, apolipoprotein^29^, and *ucp1*^30^, which are known to be PPARα signal-targeted genes containing the peroxisome proliferator response elements (PPREs) within the promoters, suggesting that cortisol activates the PPARα signalling pathway. PPARα signalling is induced by glucocorticoids in mammals^31,32^, and RNA-seq analysis using zebrafish embryos shows that gene expression related to fatty acid metabolism is changed by cortisol^33^. These results indicate that PPARα signalling, which plays an important role in fatty acid metabolism, is commonly activated by cortisol in vertebrates.

PPARα is involved in various systems such as lipid metabolism. PPARα is a nuclear receptor that binds to ligands such as fatty acids and mainly binds to the PPRE to regulate transcription^29,34^. Activation of PPARα promotes lipolysis, fatty acid oxidation, and energy uncoupling in the body^29^. PPARα is localised in gonadal somatic cells such as Sertoli, Leydig, and follicular cells in rats^34^, and our results of immunofluorescence staining showed similar localisation of Pparaa in adult medaka. Furthermore, to focus on the relationship of the expression with sexual differentiation, we investigated the localisation of Pparaa in the gonad during sexual differentiation. At 0 dph Pparaa was localised in the gonadal somatic cells in both sexes but not in the germ cells, while at 5 dph the localisation was in gonadal somatic cells and germ cells in both sexes. The expression was enhanced by cortisol at both 0 and 5 dph. Therefore, Pparaa may be activated by cortisol mainly in gonadal somatic cells during sexual differentiation.

Previously, we showed that HT and cortisol inhibited female-type proliferation of germ cells and induced the masculinisation of XX medaka^15,16^. To elucidate whether activation of PPARα induces masculinisation of XX medaka, we examined the effects of two PPARα agonists on germ cell proliferation and sex ratio. FF and GW were used as PPARα agonists in this study because the drugs have been confirmed to specifically activate PPARα in mammals and fish^24–26^. Administration of FF or GW inhibited female-type proliferation of germ cells in XX medaka during the hatching stage, and maintained males even in the adult stage. These results are similar to masculinisation by cortisol, and strongly suggest that cortisol induces masculinisation through activation of PPARα signalling. Moreover, co-administration of the agonists and cortisol increased the proportion of males compared with single administration, suggesting an interaction between PPARα and the glucocorticoid receptor^35^. Thus, these results presented for the first time the effect of PPARα activators on sexual differentiation.

Although this study indicated that activation of PPARα induces masculinisation, the molecular pathway of masculinisation mediated by PPARα was not fully elucidated. Quantitative real-time PCR showed the change in expression of sexual differentiation-related genes under cortisol treatment. Of the two gene subtypes encoding estrogen synthetase, *cyp19a1a* and *cyp19a1b*^36^, *cyp19a1b* has a higher expression level than *cyp19a1a* in the gonadal region at hatching stage, and its expression was inhibited by cortisol, suggesting that cortisol suppresses *cyp19a1b* expression through activation of PPARα during gonadal sex differentiation. In addition, in zebrafish, a PPRE exists in the promoter region of *cyp19a2* (a gene corresponding to medaka *cyp19a1b*), and the expression level of *cyp19a2* tends to be slightly reduced by treatment with PPARα agonist^37^. Therefore, PPARα may induce masculinisation by regulating directly the expression of *cyp19a1b*.

To further investigate the function of the medaka *pparaa*, we generated a *pparaa*^-/-^ medaka, which lacks the ligand-binding domain. PPARα null mice, which also lack the ligand binding domain, are viable and fertile, but are not responsive to PPARα agonists and do not increase expression of PPARα^27^ or *ucp1*^38^. *pparaa*^-/-^ medaka did not also activate PPARα signalling because the expression of *fabp7*, apolipoprotein and *ucp1* was not induced by cortisol. At the same time, in *pparaa*^-/-^ medaka, cortisol treatment did not suppress the expression of *cyp19a1b*, indicating that cortisol suppresses expression of *cyp19a1b* through activation of the PPARα signalling pathway in medaka. On the other hand, the number of germ cells at the hatching stage was not reduced by cortisol in *pparaa*^-/-^ medaka, and masculinisation of genetically female individuals did not occur in adulthood. These results demonstrate that ligand-dependent activation of PPARα signalling is important for masculinisation by cortisol. Furthermore, *pparaa*^-/-^ medaka was not masculinised by FF, suggesting that FF mainly induces masculinisation via Pparaa. Previous study shows that PPARα null mouse do not cause hepatomegaly, peroxisome proliferation, and transcriptional activation of PPARα-targeted genes by a classical peroxisome proliferator clofibrate^27^. Therefore, these fibrates may have a common PPARα-specific action from fish to mammals.

Environmental influence on sex is a topic of basic biological significance because the sex in many organisms is reversed environmentally in spite of genetic background. Environmental factors, such as pH^2^, density^3^, and social factors^4^, alter the sex ratio. Japanese flounder and medaka are masculinised by elevated cortisol due to the high temperature during sex differentiation^15,21^, while three-spot wrasses and bluehead wrasses are sex-changed by elevated cortisol due to social factors during the adult stage^22,39^. Recently, it has been reported that in bluehead wrasses, ovary-to-testis transformation involves extensive epigenetic reprogramming after elevation of cortisol^39^, suggesting an important role of cortisol in environmental sex determination and sex change. Therefore, although the masculinizing effect of PPARα signalling in other species remains uncertain, it may definitely be concluded that this signalling is involved in cortisol-induced masculinisation.

As mentioned above, PPARα mainly plays roles related to lipid metabolism and is a sensor for free fatty acids^29^. Although the relationship between reproductive function and lipid metabolism has been well studied^40^, the effects of lipid metabolism on sex determination and differentiation are poorly understood. Recently, it has been reported that fatty acid levels determine environmental sex-determination in *Caenorhabditis elegans*^41^ indicating that fatty acids may be involved in environmental sex determination in many animals. Therefore, this study provides the first evidence that lipid metabolism may be involved in environmental sex determination in vertebrates. Future studies will focus on the roles of fatty acids on environmental sex determination.

The RNA-seq and real-time PCR analyses together showed that the expression of some PPARα signalling-targeted genes was increased by cortisol. PPARα agonist treatment induced masculinisation of XX medaka in some cases, and co-treatment of the agonist with cortisol further induced masculinisation, whereas treatment of *pparaa* knockout medaka with cortisol or the agonist did not induce masculinisation, indicating that Pparaa is essential for masculinisation by cortisol. This study provides the first evidence that PPARα is involved in environmental sex determination in vertebrates. The concept of changes in lipid metabolism affecting sex can be expected to greatly contribute to artificial sex control. Further studies on the relationship between sex and lipids, such as changes in lipid metabolism and the identification of ligands and target genes for PPARα during masculinisation, are required to better understand environmental sex-determination.

## Methods

### Ethics statement

The study was performed using protocols approved by the Animal Care and Use Committee of Kumamoto University (Approval Number: 30-022). All experiments were performed in accordance with the relevant guidelines and regulations.

### Animals

The FLFII medaka stock was used^42^, which allows the identification of genotypic sex by the appearance of leucophores at 2dpf, before the onset of sex differentiation. Fish embryos and larvae were maintained in ERM (17 mM NaCl, 0.4 mM KCl, 0.27 mM CaCl_2_ 2H_2_O, 0.66 mM MgSO_4_, pH 7) at a water temperature of 26 °C in a 14 h light and 10 h dark cycle.

### Experimental treatment

HT and cortisol treatments were performed by rearing the fish at 33 °C and at 26 °C with hydrocortisone (5 × 10^–6^ M; Sigma-Aldrich, Gillingham, UK), respectively, as previously described^15,16^. PPARα agonist treatment was performed with fenofibrate (Wako, Tokyo, Japan) or GW-7647 (Tocris Bioscience, Glasgow, UK) at a concentration of 1 × 10^–6^ or 5 × 10^–6^ M from 0 dpf to 5 dph. Control was treated with 0.05% Dimethyl sulfoxide (DMSO; Sigma-Aldrich), similar to DMSO concentrations in PPARα agonist treatment, because the concentrations less than 1% do not have toxic effects for medaka embryos^43^. After treatments, fish were maintained up to adulthood (2 mph) at 26 °C. The survival rates were shown in Supplementary information Table S2.

### RNA-seq

Total RNA was extracted from the gonad regions (20 pooled samples) of XX larvae at 0 dph using NucleoSpin RNA XS (Takara Bio, Shiga, Japan) according to the manufacturer’s protocol^44^. The XX larvae untreated (control), or treated with HT or cortisol from 0 dpf to 0 dph were selected by the existence of leucophores and the gonad regions containing the guts were cut out under a stereo microscope MZ (Leica Microsystems, Wetzlar, Germany) as described previously^15^. RNA integrity was evaluated using the Agilent 2100 Bioanalyzer system (Agilent Technologies, Santa Clara, CA). An mRNA sequencing library for RNA-seq was constructed using the TruSeq RNA Library Prep kit v2 (Illumina, San Diego, CA), followed by paired-end (2 × 100 bp) sequencing using the Illumina HiSeq 4000 Sequencing System (Illumina) in the Beijing Genomics Institute. Raw paired-end reads were filtered by FASTX-Toolkit (https://hannonlab.cshl.edu/fastx_toolkit/index.html). The quality of the reads was confirmed by FastQC 0.11.3 (https://www.bioinformatics.babraham.ac.uk/projects/fastqc/). Tuxedo pipeline (Tophat software: ver. 2.0.14; Cufflinks software: ver.2.1.1) was used to map reads and to evaluate gene expression levels in FPKM^45^. Annotation of genes was based on the Hd-rR medaka assembly by National BioResource Project (https://shigen.nig.ac.jp/medaka/top/top.jsp). With the Cuffdiff analysis based on FPKM, DEGs between HT and control or between cortisol and control were identified. DEGs were screened with a false discovery rate (FDR) adjusted *p *value < 0.05. Signalling pathway matching analysis for the DEG list was performed using KEGG Mapper in Kyoto Encyclopedia of Genes and Genomes (KEGG, https://www.genome.jp/kegg/). The mapping of GO to DEGs was carried out using Blast2GO (ver. 4.1.9)^46^.

### Quantitative real-time PCR

RNA was extracted from the gonad regions of 0-dph larvae (10 pooled samples) treated with or without cortisol, using ISOGEN (Nippon Gene, Tokyo, Japan) as previously described^16^. Then, a reverse transcription was performed at 37 °C for 30 min using an RNA PCR kit (Applied Biosystems, Foster, CA). Quantitative real-time PCR was performed on a LightCycler 480 (Roche, Mannheim, Germany) using SYBR Green I Master Mix (Roche). The primers used are shown in Supplementary information Table S4. The PCR conditions were as follows: 95 °C for 5 min, then 45 cycles of 95 °C for 5 min, 59 °C for 10 s, and 72 °C for 10 s. Finally, melting curve analysis was performed after thermo-cycling to evaluate the specificity of the amplification and to verify the absence of primer dimers. Relative gene expression levels were calculated using a ΔΔ C_T_ method^47^. The RefFinder tool^48^, which integrates four specific algorithms (GeNorm^49^, NormFinder^50^, BestKeeper^51^ and the comparative delta-Ct method^52^), was used for the assessment and screening of three candidate reference genes (*elongation factor 1 alpha* (*ef1α*), *β-actin*, and *glyceraldehyde-3-phosphate dehydrogenase* (*gapdh*)). In the present study, most stably expressed *ef1a* was used as a reference gene (Supplementary information Table S3). The target genes are *peroxisome proliferator-activated receptor alpha a* (*pparaa*), *peroxisome proliferator-activated receptor alpha b* (*pparab*), *cytochrome P450, family 19, subfamily A, member 1a* (*cyp19a1a*), *cytochrome P450 family 19 subfamily A member 1b* (*cyp19a1b*), *gonadal soma-derived growth factor* (*gsdf*), *fatty acid binding protein 7* (*fabp7*), *apolipoprotein A-1b* (*apoa1b*), *apolipoprotein Bb, tandem duplicate 2* (*apobb2*), *apolipoprotein Db* (*apodb*), and *uncoupling protein 1* (*ucp1*).

### Immunofluorescence

Fish at 0 dph, 5 dph and 6 mph were fixed in 4% paraformaldehyde/PBS at 4 °C for 7 h, dehydrated, embedded in paraffin and serially sectioned at a thickness of 10 μm. Sections were incubated at 4 °C overnight with a primary antibody (1:200 dilution), rabbit polyclonal anti-Pparaa antibody (Sigma-Aldrich) against n-terminal peptide (CGDLIEDLRKISASIGDNSL) of Pparaa protein. After washing, the sections were treated for 1 h with a secondary antibody (1:200 dilution), anti-rabbit IgG conjugated with Alexa 488 (Invitrogen, Carlsbad, CA), mounted with VECTASHIELD mounting medium with DAPI (Vector Laboratories, Burlingame, CA) and then imaged with a Fluoview FV10i confocal microscope (Olympus, Tokyo, Japan).

### Histological analysis

Fish at 0 dph and 2 mph were fixed overnight at 4 °C in Bouin's solution, dehydrated, embedded in paraffin, and serially sectioned at 5 μm. Sections were stained with haematoxylin and eosin as described previously^15^. Germ cells were counted using a MZFLIII microscope (Leica Microsystems).

### Preparation and microinjection of the CRISPR/Cas9 system

Synthetic crRNA and tracrRNA were obtained from FASMAC, Co. (Kanagawa, Japan), and recombinant Cas9 protein was obtained from PNA Bio Inc. (Thousand Oaks, CA, USA) and were used as previously described^8^. The sequences of the two synthetic crRNAs for *pparaa* are shown below:

- AGCTCCTCAGACTCCCCGGCTGGguuuuagagcuaugcuguuuug,
- AATGTAGGGAGACTACAAGCAGGguuuuagagcuaugcuguuuug.

Microinjection was performed on 1-cell embryos using a Nanoject II (Drummond Scientific Co., Broomall, PA). Cas9 protein (500 ng/μL), tracrRNA (200 ng/μL) and the two crRNAs (100 ng/μL) were simultaneously injected into embryos. After injection, the embryos were maintained in ERM at 26 °C.

### Genotyping

Genomic DNA was extracted from the adult caudal fins or larval heads as described previously^15^. The genetic sex was determined by genomic PCR using primers specific for *dmy* as described previously^15^. *pparaa* genotypes were determined by PCR performed using two pairs of primers located outside and inside the defective region. PCR was performed using AmpliTaq Gold (Applied Biosystems) and the primers shown in Supplementary information Table S4.

### Statistical analysis

Experimental results were tested using Levene’s test for homogeneity of variance. Data were analysed by Student’s t-test or by one-way ANOVA followed by Turkey’s multiple comparison test using SPSS statistics 20 (IBM Corp., Armonk, NY).

## Supplementary information

Supplementary Information.
