# Supplementary material for: Peroxisome proliferator-activated receptor alpha is involved in the temperature-induced sex differentiation of a vertebrate
Source: Sci Rep. 2020 Jul 15;10:11672. doi: 10.1038/s41598-020-68594-y (PMC7363821; doi:10.1038/s41598-020-68594-y)
Supplement: Supplementary file 1 — Supplementary Information. [file 41598_2020_68594_MOESM1_ESM.pdf]

Peroxisome proliferator-activated receptor alpha is involved in the temperature-induced sex differentiation of a vertebrate

Seiji Hara[1], Fumiya Furukawa[1], Koki Mukai[1], Takashi Yazawa[2] & Takeshi Kitano[1].

[1]Department of Biological Sciences, Graduate School of Science and Technology, Kumamoto University, Kumamoto 860-8555, Japan.

[2]Department of Biochemistry, Asahikawa Medical University, Asahikawa, Hokkaido 078-8510, Japan.

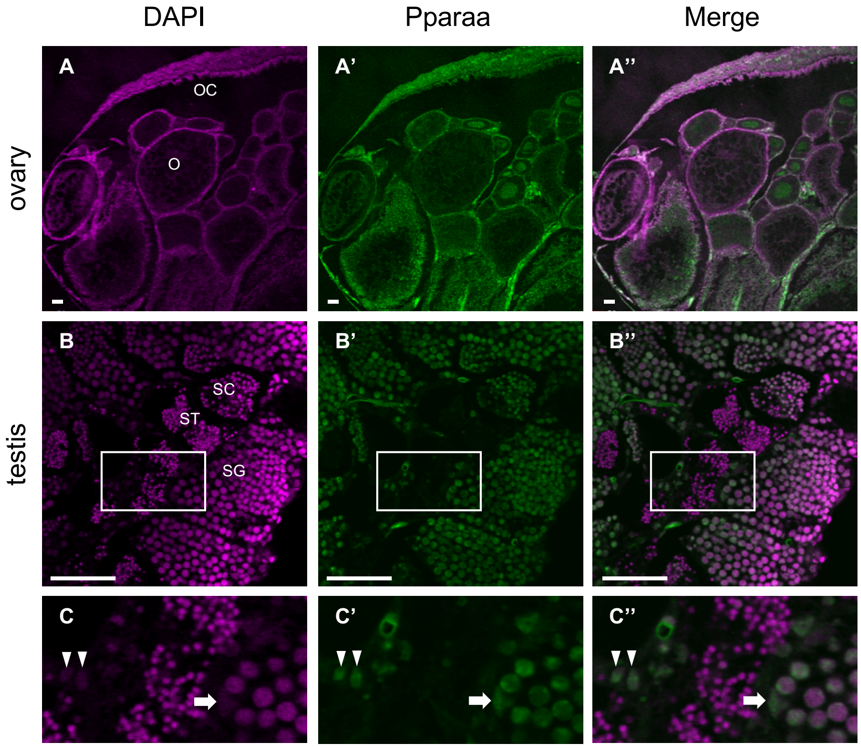

Supplementary Figure S1. Localisation of Pparaa in adult fish.

Immunofluorescence staining using an anti-Pparaa antibody. (A) 6 mph ovary, (B, C) 6 mph testis. O: oocytes, OC: ovarian cavities, SC: spermatocytes, SG: spermatogonia, ST: spermatids, arrowheads: Leydig cells, arrows: Sertoli cells. Scale bars: 50 µm.

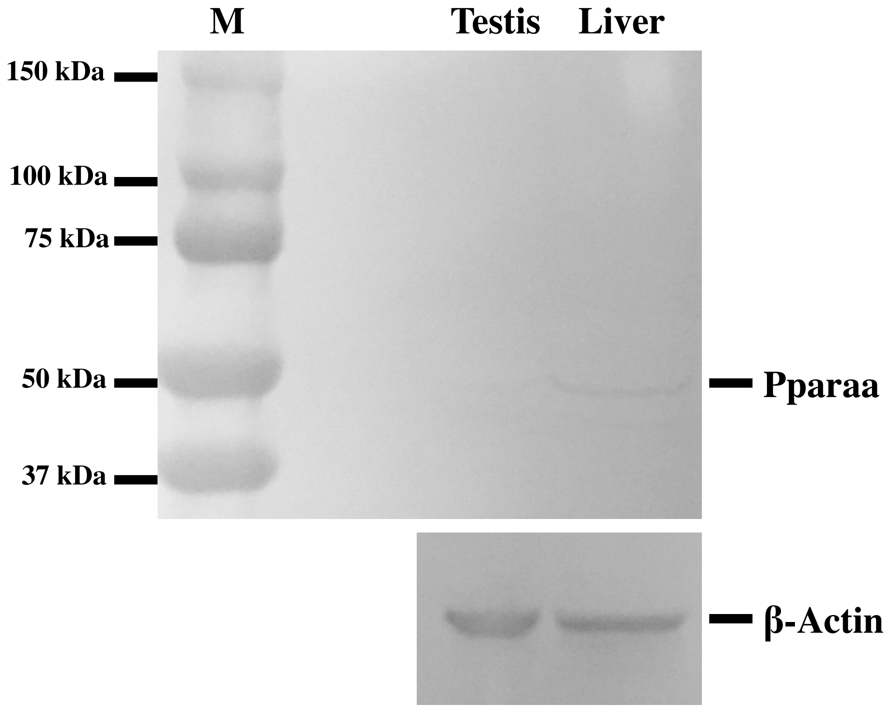

Supplementary Figure S2. Western blot analyses of Pparaa and β-Actin in adult tissues.

Protein samples extracted from the adult testis and liver were separated by SDS-PAGE and transferred to a polyvinylidene fluoride membrane as previously described[21]. The membrane was incubated at 4 °C overnight with rabbit polyclonal anti-Pparaa antibody (Sigma-Aldrich) or mouse monoclonal anti-β-actin antibody (Sigma-Aldrich) at 1:1000 dilution in Tris-buffered saline containing 5% skimmed milk, and after washing, treated for 1 h with anti-rabbit IgG or anti-mouse IgG conjugated with horseradish peroxidase at 1:2000 dilution. One-step ultra TMB-blotting solution (Thermo Fisher Scientific) was used for detection. M: protein molecular weight marker.

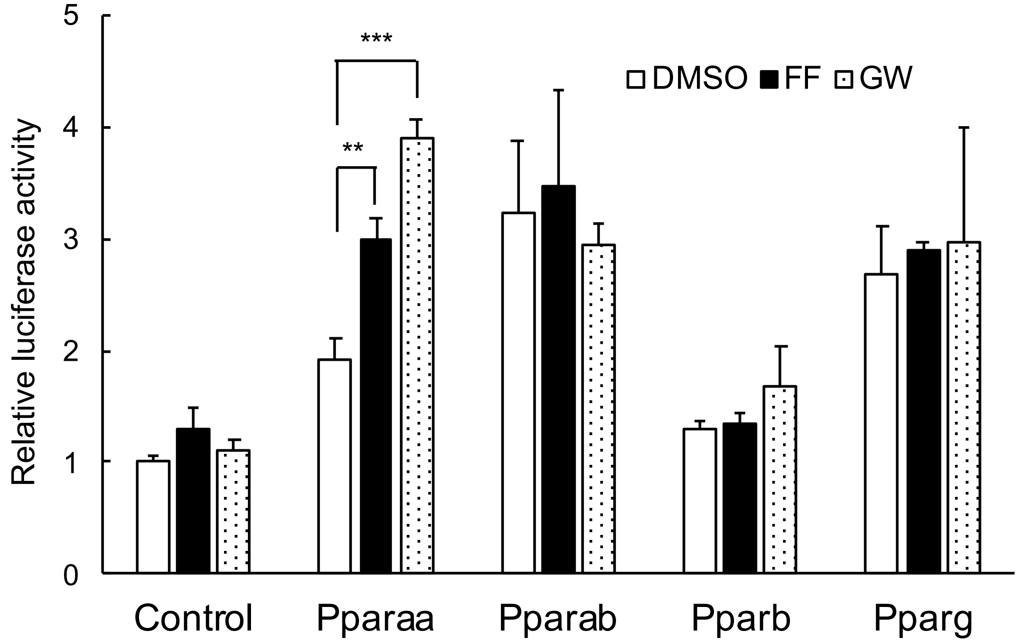

Supplementary Figure S3. Activation of medaka Ppar-mediated transcription by fenofibrate (FF) and GW-7647 (GW).

Medaka Ppar-expression vector was constructed by inserting the open reading frame of medaka Pparaa, Pparab, Pparb or Pparg into pcDNA3 (Invitrogen). The PPRE-luciferase reporter was constructed by inserting the oligonucleotides into pGL4.24 (Promega). Medaka hepatoma OLHE-131 cells were cultured in L-15 medium supplemented with 10% fetal bovine serum at 28°C. Cells were plated in 48-well plates 24 h before transfection. Cells were transfected with 400 ng of the PPRE-luciferase reporter, 50 ng of medaka Ppar-expression vector or empty vector (control), and 50 ng of pRL-TK *Renilla* luciferase normalisation vector (Nippon Gene) using FuGENE 6 (Roche) as described previously[15]. After 24 h of transfection, the cells were treated with vehicle (DMSO), fenofibrate (50 μM) or GW-7647 (10 μM) for 24 h. Luciferase assays were performed as described previously[15]. Values of controls treated with DMSO were defined as 1. Data represent the mean ± SEM of three independent experiments. $^{**}p < 0.01$. $^{***}p < 0.001$.

Supplementary Table S1. List of GO terms related to lipids among the DEGs.

up-regulated

Ontology	Description	Accession No.	e-value
P:lipid metabolic process	patatin-like phospholipase domain-containing 2	ENSORLG00000006274	1.25E-49
P:lipid metabolic process	lipocalin-like	ENSORLG00000006624	3.13E-64
P:lipid metabolic process	lipocalin-like	ENSORLG00000007143	4.69E-69
P:lipid metabolic process	group XIIB secretory phospholipase A2 isoform X2	ENSORLG00000011386	1.21E-92
P:lipid transport	apolipo B-100-like	ENSORLG00000012638	0
P:lipid transport	apolipo B-100-like	ENSORLG00000012653	0
F:lipid binding	apolipo D-like	ENSORLG00000013153	2.57E-52
F:lipid binding	apolipo A-IV	ENSORLG00000015744	5.23E-135
F:lipid transporter activity	apolipo B-100-like	ENSORLG00000012638	0
F:lipid transporter activity	apolipo B-100-like	ENSORLG00000012653	0

down-regulated

Ontology	Description	Accession No.	e-value
F:lipid binding	apolipo D-like	ENSORLG00000017942	1.01E-49
F:lipid binding	apolipo D-like	ENSORLG00000017946	5.13E-91
P:lipid metabolic process	galactosylceramide sulfotransferase-like	ENSORLG00000008121	0
P:lipid metabolic process	phospholipase A2-like	ENSORLG00000018361	2.83E-25

Supplementary Table S2. Hatching and survival rates during chemical treatment.

Genotype	Treatment	Hatching rate (No. of hatching fish / No. of eggs)	Survival rate at 5 days post-hatching (No. of surviving fish / No. of hatching fish)
wild-type	Control	90.1 % (73/81)	88.2 % (60/68)
	Cortisol	89.6 % (43/48)	88.9 % (104/117)
	1 μM FF	80.5 % (62/77)	87.3 % (62/71)
	5 μM FF	80.4 % (74/92)	73.0 % (54/74)
	5 μM FF + Cortisol	0 % (0/29)	91.7 % (66/72)
	1 μM GW	91.2 % (52/57)	68.6 % (24/35)
	1 μM GW + Cortisol	-	62.3 % (43/69)
pparaa$^{-/-}$	Control	96.4 % (53/55)	86.4 % (38/44)
	Cortisol	61.4 % (35/57)	96.6 % (28/29)
	5 μM FF	-	49.2 % (31/63)

Supplementary Table S3. Expression stability of reference genes determined by RefFinder.

	Ranking Order (Better - Good - Average)		
Method	1	2	3
⊿Ct	ef1α	gapdh	β-actin
BestKeeper	ef1α	β-actin	gapdh
NormFinder	ef1α	gapdh	β-actin
geNorm	ef1α & gapdh		β-actin
total	ef1α	gapdh	β-actin

Supplementary Table S4. List of primers used in this study.

Purpose	Primer name	Sequence (5' - 3')	Accession #
Quantitative real-time PCR	pparaa-S	ACCCCTTAACTCCAGCCTCC	AB469412
	pparaa-AS	CTCCAGCTTCAGCCTGATGG	
	pparab-S	GTAGCCACCATCATCTGCTG	AB469411
	pparab-AS	GTCTCGGTAGATCTCCTGTAG	
	fabp7-S	CCTTCAACATGGTTGACGCC	AB127930
	fabp7-AS	CTCTGACGTGCTTGGTCTCC	
	gsdf-S	ATGTCTTTGGCACTCATTGTC	AB525390
	gsdf-AS	ACTGTCGCATGACACAGAGG	
	cyp19a1a-S	ATGGATCTGATCCCTGCTTG	AB030455.1
	cyp19a1a-AS	CCACACTCGAACAATGTCTC	
	cyp19a1b-S	CTGGCCCAAGCCTTCAGAGG	AB591736
	cyp19a1b-AS	CAATCGCTCTGAGGAGATTG	
	apoa1b-S	GCACATGGACTCAAGCCTCC	CP020677.1
	apoa1b-AS	CATGTAGACATCTGCAGCGG	
	apobb2-S	GGTTCTTCCCAGACACCACC	CP020688.1
	apobb2-AS	CTGGAGACTCTGCAGCCTTC	
	apodb-S	AGGCGAACTACTCCCTGAGG	CP020684.1
	apodb-AS	CAGAGAGCCGTGAATGGAGG	
	ucp1-S	GTTGTACAACGGGCTGGTGG	CP020665.1
	ucp1-AS	TCAGGTTCATCTGGGCCTGG	
	ef1α-S	TGAGATGGGCAAGGGCTCCT	AB013606
	ef1α-AS	GCTGGGTTGTAGCCGATCTT	
	β-actin-S	TCCACCTTCCAGCAGATGTG	S74868
	β-actin-AS	AGCATTTGCGGTGGACGAT	
	gapdh-S	CCTCCATCTTTGATGCTGGT	CP020680.1
	gapdh-AS	ACGGTTGCTGTAGCCAAACT	
Genotyping	pparaa-outer-S	CGTTTCAGCTAGCTGAGAGG	AB469412
	pparaa-outer-AS	GGTTATTGTTTTCTTTACACAG	
	pparaa-inner-S	CCTGGGATTTAAACGATCAGG	
	pparaa-innner-AS	CCGCATATTTCAGAAGGGTC	
	dmy-S	GTCAAGGCAGAGTTTGAGAG	AB071534
	dmy-AS	CATTGTGACCAACATCTTCTG	
